# Advances in the Regulation of Macrophage Polarization by Mesenchymal Stem Cells and Implications for ALI/ARDS Treatment

**DOI:** 10.3389/fimmu.2022.928134

**Published:** 2022-07-08

**Authors:** Chang Liu, Kun Xiao, Lixin Xie

**Affiliations:** ^1^ School of Medicine, Nankai University, Tianjin, China; ^2^ Center of Pulmonary & Critical Care Medicine, Chinese People’s Liberation Army (PLA) General Hospital, Beijing, China; ^3^ Medical School of Chinese People’s Liberation Army (PLA), Beijing, China

**Keywords:** macrophage polarization, mesenchymal stem cells, acute lung injury, acute respiratory distress syndrome, treatment

## Abstract

Acute lung injury/acute respiratory distress syndrome (ALI/ARDS) is a common condition with high mortality. ALI/ARDS is caused by multiple etiologies, and the main clinical manifestations are progressive dyspnea and intractable hypoxemia. Currently, supportive therapy is the main ALI/ARDS treatment, and there remains a lack of targeted and effective therapeutic strategies. Macrophages are important components of innate immunity. M1 macrophages are pro-inflammatory, while M2 macrophages are anti-inflammatory and promote tissue repair. Mesenchymal stem cells (MSCs) are stem cells with broad application prospects in tissue regeneration due to their multi-directional differentiation potential along with their anti-inflammatory and paracrine properties. MSCs can regulate the balance of M1/M2 macrophage polarization to improve the prognosis of ALI/ARDS. In this paper, we review the mechanisms by which MSCs regulate macrophage polarization and the signaling pathways associated with polarization. This review is expected to provide new targets for the treatment of ALI/ARDS.

## 1 Introduction

### ALI/ARDS

Acute lung injury (ALI) and its more severe form, acute respiratory distress syndrome (ARDS), are critical illnesses caused by excessive and uncontrolled systemic inflammatory responses to direct or indirect lung injury. ALI is defined as the acute onset of diffuse bilateral pulmonary infiltrates by chest radiograph, and with a PaO2/FiO2 ≤ 300mmHg or without clinical evidence of left atrial hypertension (1). Those with more severe hypoxemia (PaO2/FiO2 ≤ 200mmHg) are considered to have ARDS. While according to the Berlin definition, ARDS is divided into three categories in terms of the degree of hypoxemia: mild (PaO_2_/FiO_2_ = 200–300 mmHg); moderate (PaO_2_/FiO_2_ = 100–200 mmHg); and severe (PaO_2_/FiO_2_ < 100 mmHg) (2). ALI and ARDS are characterized by severe intractable hypoxemia, hypoxic respiratory failure, alveolar and interstitial pulmonary edema, decreased pulmonary compliance, and increased pulmonary vascular permeability (3, 4). The main pathological changes associated with ALI/ARDS include damage to alveolar epithelial cells and alveolar capillary endothelial cell barriers, the exudation of protein-rich edema fluid from the alveolar lumen, and the infiltration of inflammatory cells such as neutrophils and macrophages (5).

ALI/ARDS may be caused by a variety of factors including shock, severe sepsis, pulmonary contusion, gastroesophageal reflux, aspiration of gastric contents, pneumonia, drug toxicity, blood transfusion, acute pancreatitis, ischemia-reperfusion, and drowning (6).

The pathogenesis of ALI/ARDS is complex, and the main pathogenic mechanisms are excessive inflammatory response caused by the massive release of pro-inflammatory cytokines and damage to alveolar epithelial cells resulting from the excessive activation of multiple immune cells. The lung tissue can be stimulated by multiple factors to trigger infiltration of various inflammatory cells such as macrophages and neutrophils, thereby releasing large amounts of pro-inflammatory cytokines and inflammatory mediators. This disrupts the integrity of alveolar epithelial cells and the pulmonary capillary endothelial cell barrier, leading to pulmonary edema and alveolar hemorrhage (6, 7). ARDS can be classified into two subtypes, hyperinflammatory and hypoinflammatory, based on clinical features such as oxygenation index and inflammation-related biomarkers such as the levels of IL-6, IL-8, IL-18, TNF-α, and ACE2. The hyperinflammatory subtype of ARDS has poor prognosis and high mortality (8).

Treatment of ALI/ARDS is supportive. The current clinical treatment of ALI/ARDS is based on lung-protective mechanical ventilation and fluid management therapy (9), supplemented by glucocorticoids (10, 11), surfactants (12, 13), N-acetylcysteine (14, 15), statins (16, 17), β2 agonists (18), neuromuscular blockade (3, 19), extracorporeal membrane pulmonary oxygenation (20) and prophylaxis for venous thromboembolism (21). In addition, for infections such as sepsis-induced ARDS, antimicrobial drugs can be used pertinently (22). Moreover, for ARDS caused by blood transfusion, the dose-response relationship between the amount of blood products transfused in patients and the risk of developing ARDS suggested that restrictive transfusion policies may reduce the incidence of ARDS in these patients (23, 24). Although these supportive treatments can improve patients’ symptoms to a certain extent, they do not significantly improve the prognosis, and the associated mortality remains high. An international multicenter prospective cohort study indicated that the intensive care unit (ICU) admission of ARDS patients was 10.4%, with an overall mortality rate of 35%–46% (25). Another large clinical trial found a 43% mortality rate at 90 days in patients with ARDS (26). Even patients in recovery may face long-term cognitive impairment and impaired quality of life (27). Moreover, although mechanical ventilation is the basic and important methods for the treatment of ALI/ARDS, long-term/excessive mechanical ventilation may lead to ventilator-related lung injury (VILI) and even pulmonary fibrosis (28–33). Therefore, there is an urgent need to explore more effective and safe therapeutic measures for ALI/ARDS.

### Mesenchymal Stem Cells (MSCs)

MSCs are pluripotent, self-renewing stem cells with multi-directional differentiation potential and migration ability. MSCs originate from a variety of organs and tissues such as bone marrow, adipose, muscle, umbilical cord, and placenta tissues. Based on these characteristics, MSCs are widely used in the field of tissue regeneration (34, 35). MSCs can regulate immune homeostasis, reduce lung inflammation, repair tissue damage, and promote tissue regeneration, making them promising for the treatment of ALI/ARDS (36–39).

### Macrophages

The presence of macrophages in lung tissue plays a critical role in the inflammatory response to ALI/ARDS. Macrophages in lung tissue mainly include two subpopulations: alveolar macrophages and interstitial macrophages. Alveolar macrophages, which are more abundant than interstitial macrophages, are the first line of defense against foreign invading factors and play an important role in host defense and the maintenance of immune homeostasis in the local microenvironment of lung tissue (40–42). In response to stimulation by foreign pathogens, alveolar macrophages can release a variety of inflammatory factors and chemokines to initiate a cascade of amplified inflammatory responses within lung tissue and mediate lung tissue injury.

**Figure 1:**
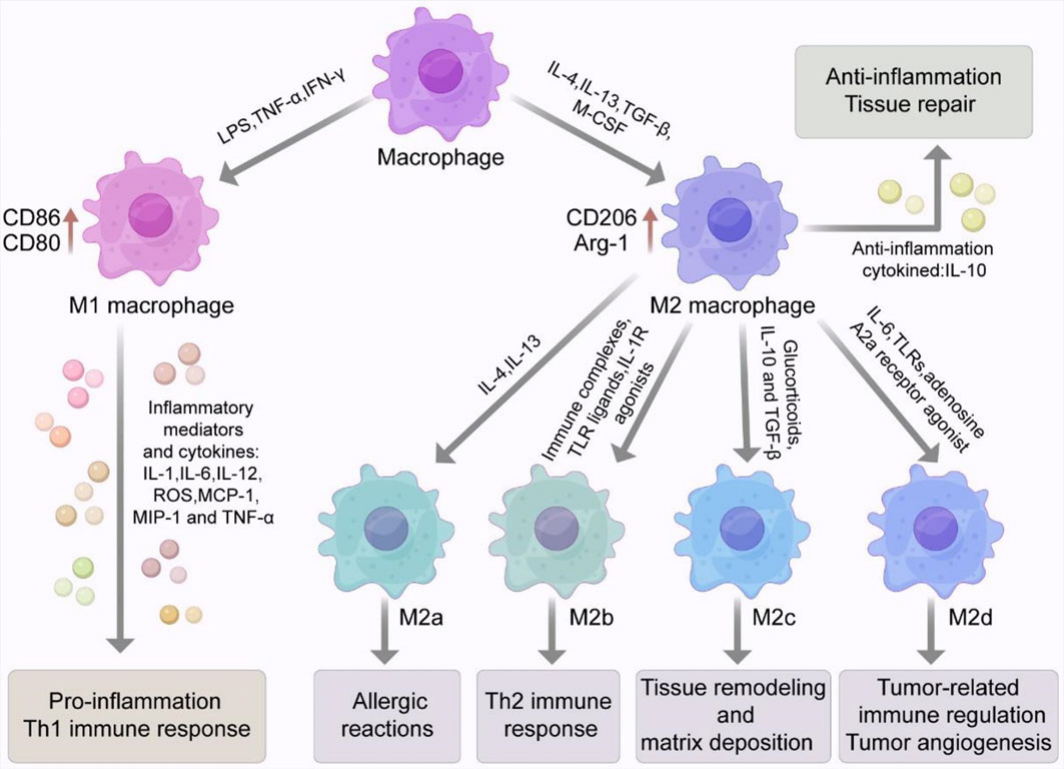
Macrophage phenotype and polarization. Macrophages have good plasticity and can differentiate into two phenotypes, classically activated M1 macrophages or alternatively activated M2 macrophages under different environmental conditions. Stimulated by LPS or Th1 related cytokines such as IFN-γ and TNF-α, macrophages can polarize into the M1 phenotype with high expression of surface markers such as CD86 and CD80. M1 macrophages can secrete a variety of pro-inflammatory cytokines and inflammatory mediators that can cause a strong inflammatory response and cause tissue damage. M2 macrophages are usually induced by IL-4, IL-13, TGF-β and M-CSF. M2 macrophages highly express specific surface markers such as CD206, CD163, Arg-1, Ym-1 and Fizz 1. M2 macrophage also secrete a large amount of the anti-inflammatory cytokine IL-10, which inhibits inflammatory responses and participates in tissue repair. M2 macrophages can be divided into four subtypes (M2a, M2b, M2c, M2d). Activation of M2a macrophages is induced by IL-4 and IL-13 and may be involved in allergic reactions. M2b macrophages are induced by immune complexes (ICs), Toll-like receptor (TLR) ligands and IL-1R agonists. Glucocorticoids, IL-10 and TGF-β are involved in the induction of M2c macrophages and may be involved in tissue remodeling and stromal deposition. M2d macrophages are induced synergistically by TLR and adenosine A2a receptor agonists or IL-6. With the characteristics of tumor-like macrophages, they can be involved in tumor-associated immune regulation and tumor angiogenesis.

Macrophages have good plasticity and can polarize into different phenotypes under different environmental conditions, including classically activated M1 macrophages and alternatively activated M2 macrophages (**Figure 1**). M1 macrophages produce a large amount of pro-inflammatory cytokines, which cause tissue damage while facilitating the host immune clearance of pathogens. M2 macrophages mainly secrete anti-inflammatory cytokines, which facilitate wound healing and the repair of tissue damage (43–45).

The M1 and M2 phenotypes of macrophages can be interconverted under certain conditions. M1 and M2 macrophages can effectively avoid excessive inflammatory responses that cause tissue damage by maintaining the immune homeostasis of the lung microenvironment. In the progression of ALI/ARDS, the balance of M1 and M2 macrophages can effectively remove harmful substances and over-produced pro-inflammatory cytokines from the body to promote the repair of lung tissue damage. However, the loss of balance may exacerbate lung injury and worsen ALI/ARDS. MSCs can modulate macrophage function by regulating the polarization of macrophages; thus, MSCs show promise for the treatment of ALI/ARDS and the improvement of ALI/ARDS patient prognosis (46–50).

## 2 Macrophage Phenotypes and ALI/ARDS

### M1 Macrophages and ALI/ARDS

Macrophages are usually polarized to the M1 phenotype in response to microbial stimuli including lipopolysaccharide (LPS) and Th1-related cytokines such as IFN-γ and TNF-α (44) (**Figure 1**). M1 macrophages highly express CD86, CD80, and other surface markers. M1 macrophages also secrete numerous inflammatory mediators and cytokines such as TNF-α, IL-1, IL-6, IL-12, iNOS, reactive oxygen species (ROS), monocyte chemotactic protein 1 (MCP-1), and macrophage inflammatory protein 2 (MIP-2). These compounds lead to a strong inflammatory response and Th1 immune response and cause tissue damage while participating in pathogen clearance (51). In the early stage of ALI/ARDS, alveolar macrophages are M1-polarized and release various pro-inflammatory factors and harmful mediators while clearing pathogenic microorganisms and recruiting neutrophils and other inflammatory cells. Eventually, the excessive accumulation of pro-inflammatory cytokines and inflammatory cells lead to lung tissue injury (52).

### M2 Macrophages and ALI/ARDS

M2 macrophages are usually induced by IL-4, IL-13, TGF-β, and macrophage colony-stimulating factor (M-CSF; **Figure 1**). Activated M2 macrophages highly express arginase 1 (Arg-1) and specific surface markers such as CD206, CD163, chitinase 3-like 3 (Ym-1) and found in inflammatory zone 1 (Fizz1). M2 macrophages also secrete large amounts of the anti-inflammatory cytokine IL-10, which inhibits inflammatory cell aggregation and negatively regulates the production of anti-inflammatory factors (53).

M2 macrophages can be further divided into four subtypes: M2a, M2b, M2c, and M2d (**Figure 1**). The activation of M2a macrophages is induced by IL-4 and IL-13, which can be involved in allergic reactions, wrapping and killing parasites. M2b macrophages are induced by immune complexes (ICs), Toll-like receptor (TLR) ligands, and IL-1R agonists. M2b macrophages, which secret high levels of IL-10 and low levels of IL-12, which is conductive to the Th2 immune responses. Glucocorticoids, IL-10, and TGF-β are involved in the induction of M2c macrophages. Activated M2c macrophages negatively regulate the production of anti-inflammatory cytokines involved in tissue remodeling and matrix deposition (43, 44, 54–56). M2d macrophages are synergistically induced by TLRs and adenosine A2a receptor agonists or by IL-6. M2d macrophages have the characteristics of tumor-like macrophages and can participate in tumor-related immune regulation, tumor growth, and tumor angiogenesis (57–59). In addition, some investigators also described a new subpopulation of pro-resolving macrophages, namely CD11b^low^ macrophages that emerge during the resolution of zymosan-induced peritonitis in mice. These macrophages secrete pro-resolving mediators and are generated by M2-like macrophages *in vivo* and *in vitro* after phagocytosis of apoptotic leukocytes (60). Another study suggested that pro-resolving macrophages could produce antiangiogenic mediators such as endostatin, which could halt angiogenesis and restore tissue structure (61). In summary, macrophages are highly plastic cells. They may exert a vast “spectrum” of functions characterized by an array of different macrophage phenotypes/subtypes (62).

M2 macrophages are key regulators of tissue repair during ALI/ARDS recovery (63, 64). M2 macrophages can release anti-inflammatory cytokines, inhibit the production of pro-inflammatory mediators, and remove apoptotic neutrophils from inflammatory sites to promote lung injury repair. M2 macrophages can also suppress the expression of iNOS and prevent the production of ROS, thereby reducing damage to alveolar epithelial cells and promoting the recovery of host tissues (52, 65–67).

In summary, M2 macrophages can suppress inflammation, promote tissue remodeling, and participate in angiogenesis and immune regulation. M2 macrophages are also thought to promote fibrosis. The TGF-β1 secreted by M2 macrophages can trigger fibroblast activation and extracellular matrix-producing myofibroblast development while promoting the resolution of inflammatory response to drive fibrosis (68, 69). Maintaining the appropriate balance of M1 and M2 macrophages *in vivo* thus provides a target for the treatment of ALI/ARDS.

## 3 MSCs Modulate Macrophage Polarization to Attenuate ALI/ARDS

MSCs can regulate the transition of macrophages to play an important role in ALI/ARDS. In a model of ALI caused by sulfur mustard, treatment with adipose-derived MSCs decreased the expression of CD86 markers by macrophages, suggesting that MSCs inhibited macrophage polarization to the pro-inflammatory phenotype and promoted polarization to the anti-inflammatory phenotype (70). A study found that heat shock-pretreated umbilical cord-derived MSCs (UC-MSCs) exhibited enhanced immunomodulatory effects by inducing anti-inflammatory macrophage polarization (49). Moreover, the levels of M1 markers were significantly elevated after LPS precondition, while co-culturing with heat shock-pretreated UC-MSCs reversed this effect. This may be because heat shock pretreatment enhanced the protein levels of HSP70 in UC-MSCs and negatively regulated the activation of inflammasomes in macrophages (49). Another study found that a stress response protein with anti-oxidant capacity was highly expressed in MSCs; the knocking down of this protein decreased IL-10 secretion and diminished anti-inflammatory macrophage polarization (71).

Dental follicle stem cells, a unique population of MSCs, were found to attenuate histopathological damage and lung permeability, downregulate pro-inflammatory cytokines such as MCP-1, IL-6, and TNF-α, upregulate the anti-inflammatory cytokine IL-10, increase the expression of the anti-inflammatory macrophage marker Arg-1, and decrease the production of the pro-inflammatory macrophage polarization markers iNOS and CD86 (72). Morrison et al. found that co-culturing macrophages stimulated with bronchoalveolar lavage fluid (BALF) from ARDS patients with human bone marrow MSCs (BMSCs) increased the expressions of anti-inflammatory macrophage markers (73). The above studies demonstrate that MSCs can attenuate lung histopathological changes and the inflammatory response in the ARDS inflammatory microenvironment by regulating the balance between pro-inflammatory and anti-inflammatory macrophages.

## 4 How MSCs Regulate Macrophage Polarization

### MSCs Regulate Macrophage Polarization Through the Paracrine Effects of Soluble Factors

**Figure 2:**
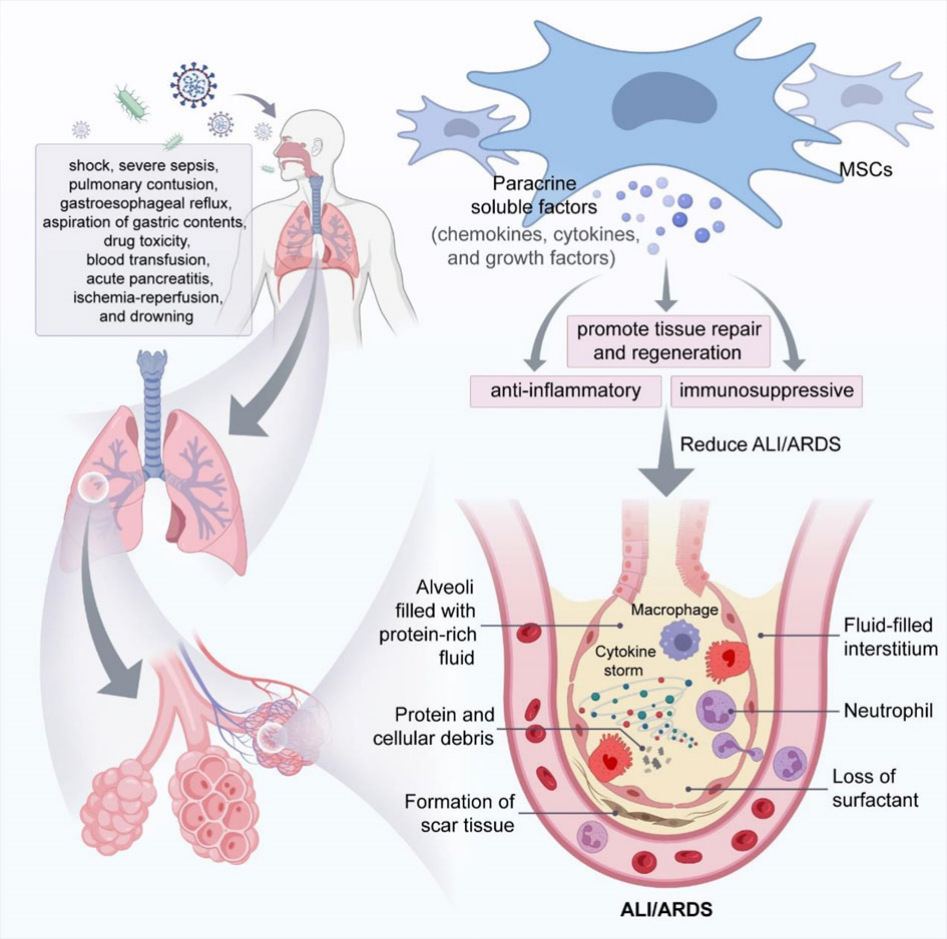
MSCs attenuate ALI/ARDS through paracrine soluble factors. In the phase of ALI/ARDS, immune cells (e.g., macrophages and neutrophils) accumulate in the alveolar space and produce large amounts of cytokines, leading to a cytokine storm that will eventually cause decreased surfactant from alveolar epithelial cells and fluid accumulation in the alveolar space and interstitium, resulting in alveolar and pulmonary interstitial edema, and MSCs can play a key role in mitigating ALI/ARDS through the immunomodulatory effects of paracrine soluble factors.

MSCs can home to injured sites to reduce inflammatory response. MSCs differentiate toward type II alveolar epithelial cells, which participate in the tissue repair process (74). However, some studies suggested MSCs retained a low number and had a short duration in the injured tissue after transplantation (75–79). Therefore, MSCs homing and differentiation are not the key mechanisms of their effects in ALI/ARDS. Rather, paracrine action may be the primary mechanism of MSCs involvement in tissue injury and repair (**Figure 2**).

MSCs secret soluble factors such as chemokines, cytokines, and growth factors, which exert paracrine effects to promote tissue repair and regeneration (80, 81). The paracrine action of MSCs plays a crucial anti-inflammatory and immunosuppressive role in immune cells. Wakayama et al. reported that MSCs promoted macrophage polarization to the anti-inflammatory phenotype *via* a paracrine mechanism, expressed high levels of CD206 and Arg-1, attenuated pro-inflammatory responses, and reduced bleomyclin-induced ALI in mice (82).

MSCs from dental capsules promoted anti-inflammatory polarization *via* the paracrine secretion of TGF-β3 and thrombospondin-1 to upregulate the level of anti-inflammatory factors, thereby reducing the pulmonary inflammatory response (72). Kwon et al. suggested that UC-MSCs could regulate the anti-inflammatory response by secreting decorin, a key regulator that polarizes inflammatory macrophages into anti-inflammatory macrophages (83). Kim et al. reported that the paracrine secretion of Pentraxin 3 from UC-MSCs enhanced the levels of anti-inflammatory macrophage markers and anti-inflammatory cytokines to improve hyperoxic lung injury (84). MSCs also attenuated lung inflammation by promoting the anti-inflammatory macrophage phenotype *via* the paracrine secretion of insulin-like growth factor (85). Prostaglandin (PGE2) secreted by MSCs induced an immunosuppressive anti-inflammatory phenotype in alveolar macrophages, increased the ability to produce IL-10, and inhibited the production of TNF-α (86). Other soluble factors such as Galectin-9 (87), tumor necrosis factor-stimulated gene 6 (TSG-6) (88), indoleamine 2,3-dioxygenase (89), and IL-6 (90) may also be involved in the regulation of macrophage polarization to combat inflammation.

### MSCs Regulate Macrophage Polarization *via* Their Derived Exosomes

**Figure 3:**
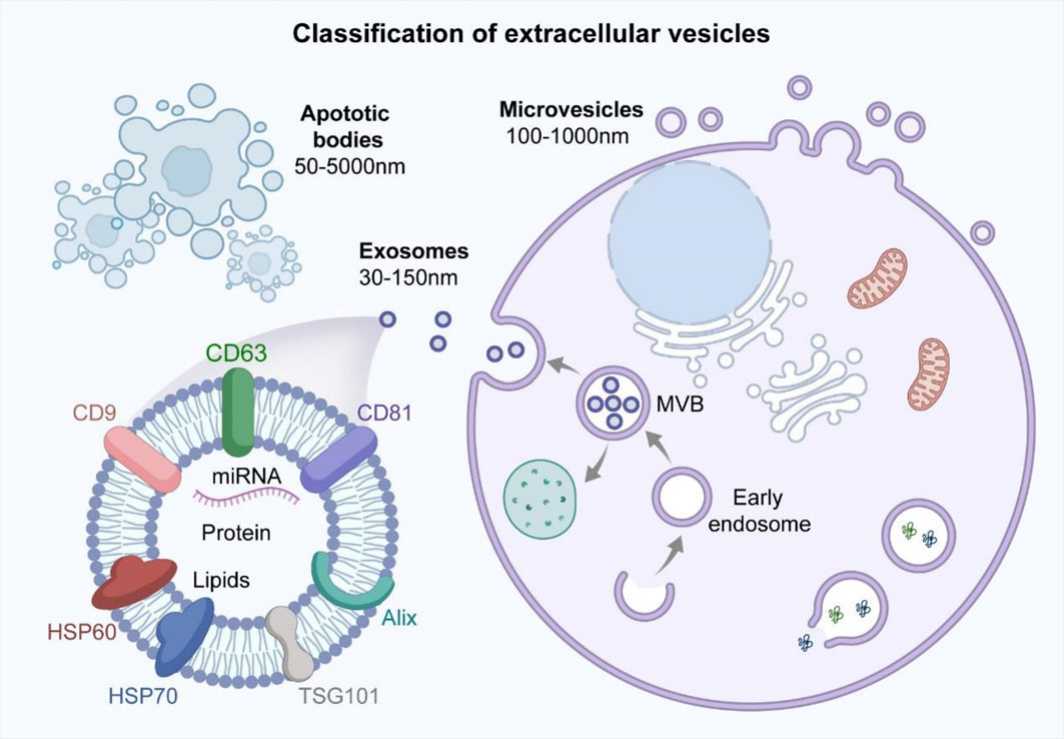
Classification of extracellular vesicles and structural characteristics of exosomes. Extracellular vesicles can be classified into three main subtypes based on biological properties and diameter size: exosomes (30-150nm), microvesicles (100-1000nm) and apoptotic bodies (50-5000nm). The surface of exosomes contains specific protein markers such as tetraspanins (CD9, CD81, CD63), HSP60, HSP70, TSG101 and Alix. Exosomes carry proteins, lipids and miRNA that are involved in mediating intercellular communication and regulating cellular activities, performing a variety of biological functions.

MSC-derived extracellular vesicles can be divided into three main subtypes based on their biological properties and diameters: exosomes (30–150 nm), microvesicles (100–1000 nm), and apoptotic vesicles (50–5000 nm) (91–94) (**Figure 3**). Exosomes are produced through the intracellular body pathway as follows. The cell membrane is invaginated to form early endosomes, which interact with vesicles formed by Golgi apparatus budding to form late endosomes. The late endosomes further develop into multivesicular bodies (MVBs) that contain intracellular vesicles. The MVBs fuse with the lysosomal membrane or cell membrane and degrade, releasing the contents (i.e., exosomes) into the extracellular environment through exocytosis (95, 96). The process of exosome formation is shown in **Figure 3**. The endosomal sorting complex required for transport plays a key role in exosome biogenesis (97). The density of exosomes is approximately 1.13–1.19 g/ml, and exosomes are widely found in plasma, serum, urine, BALF, breast milk, saliva, tears, ascites, and other body fluids (98–104). Exosomes express tetraspanins (CD9, CD81, CD63, and CD82), TSG10, heat-shock proteins, and apoptosis-linked gene 2-interacting protein X (Alix) (105–107). Exosomes carry proteins, lipids, and RNAs that mediate intercellular communication between different cell types, participate in the regulation of cellular activity, and perform a variety of biological functions (**Figure 3**) (108–111). Moreover, exosomes derived from MSCs can act as carriers of transported miRNA, enabling communication with recipient cells; thus, MSC-derived exosomes can affect disease pathogenesis along with tissue repair and regeneration (112, 113).

MSC-derived exosomes can attenuate ALI/ARDS by regulating macrophage polarization. Tian et al. found that adipose MSC-derived exosomes inhibited TLR4 expression to promote macrophage polarization and attenuate septic lung injury in mice (114). Song et al. reported that miR-146a expression was upregulated in MSC-derived exosomes after IL-1β stimulation, leading to macrophage polarization into the anti-inflammatory phenotype and increased survival in mice with sepsis-induced lung injury (115). Bao et al. demonstrated that MSC-derived exosomes can regulate alveolar macrophage pro-inflammatory polarization and block the pro-inflammatory pathway of macrophages, thereby reducing radiation-induced lung injury in mice (116). Lastly, Li et al. reported that the intratracheal administration of MSC-derived exosomes decreased pro-inflammatory polarization in alveolar macrophages while increasing anti-inflammatory polarization, leading to reduced pulmonary edema and pulmonary dysfunction along with decreased secretion of inflammatory factors such as IL-1β, IL-6, and TNF-α (117).

### MSCs Regulate Macrophage Polarization Through Metabolic Reprogramming

Macrophage polarization is closely related to metabolic status. Pro-inflammatory macrophage polarization is characterized by enhanced glycolysis and pentose phosphate pathways and decreased oxidative phosphorylation. In contrast, anti-inflammatory macrophage polarization is characterized by enhanced oxidative phosphorylation, and anti-inflammatory macrophages rely on oxidative phosphorylation for energy production (118–121). MSCs can induce anti-inflammatory macrophage polarization and promote tissue repair through metabolic reprogramming (122). Deng et al. found that BMSC-derived exosomes inhibited pro-inflammatory polarization and promoted anti-inflammatory polarization in mice alveolar macrophages (63). The authors further demonstrated that BMSC-derived exosomes reduced LPS-induced lung pathological injury by inhibiting the expression of hypoxia-inducible factor 1-α and downregulating the expressions of key proteins of macrophage glycolysis, thereby regulating macrophage polarization and inflammatory response.

### MSCs Regulate Macrophage Polarization Through Mitochondrial Transfer

In addition to increased lung epithelial and endothelial barrier permeability, the inflammatory response of ALI/ARDS can also cause mitochondrial dysfunction in lung tissue (123). MSCs may provide mitochondria to damaged cells, thereby enhancing cellular bioenergetics and improving organ dysfunction in ALI/ARDS and other inflammatory diseases. MSCs have been shown to restore mitochondrial function and alveolar bioenergetics through functional mitochondrial transfer, promote pulmonary epithelial injury repair, and improve alveolar capillary barrier permeability (124, 125).

Jackson et al. found that the extensive mitochondrial transfer of MSCs to macrophages enhanced macrophage phagocytosis and played an antibacterial role in a model of lung injury caused by *E. coli*-induced pneumonia (126). The mitochondrial transfer of MSCs plays a key role in regulating macrophage phenotype; Morrison et al. found that MSCs promoted macrophage oxidative phosphorylation through extracellular vesicle-mediated mitochondrial transfer and increased the expression of the anti-inflammatory macrophage marker CD206, thereby converting macrophages into the anti-inflammatory phenotype and ameliorating lung injury (73). Moreover, MSCs-derived exosomes can donate mitochondria components, improve macrophage mitochondrial integrity and oxidative phosphorylation levels, transform macrophages into an anti-inflammatory phenotype, and restore the metabolic and immune homeostasis of alveolar macrophages and reduce lung inflammation (127).

### MSCs Regulate Macrophage Polarization Through Apoptotic and Efferocytosis Effects

MSCs undergo necessary apoptosis and releases apoptotic vesicles during therapeutic application, apoptotic MSCs have unique anti-inflammatory properties and immunomodulatory effects (128). Apoptotic umbilical cord MSCs were able to reduce inflammatory exudates and vascular permeability in ALI rat lungs more effectively than normal umbilical MSCs. It could also more effectively reduce the level of pro-inflammatory factors and could more effectively increase the expression of anti-inflammatory cytokine IL-4, which was able to reduce the degree of pathological damage in rats with ALI (129). In addition, apoptotic MSCs could also exert a beneficial role in sepsis and allergic airway inflammation (130, 131).

Macrophages and monocytes that phagocytose apoptotic cells are capable of producing anti-inflammatory mediators (132, 133). When graft-versus-host disease patients were infused with *in vitro*-generated apoptotic MSCs, it was observed that recipient phagocytes engulfed the apoptotic MSCs and produced indoleamine 2,3-dioxygenase, thereby achieving an immunosuppressive effect (134).

In an animal model of ovalibumin-induced allergic asthma, the investigators showed that apoptotic MSCs exerted immunosuppressive effects in the lungs and inhibited allergic asthma to a similar extent as administration with viable MSCs (135). Besides, apoptotic MSCs inhibited the inward flow of eosinophils in BALF and the production of specific IL-5 and IL-13 in ovalbumin-induced allergic asthma to a similar extent as surviving MSCs, which indicated that the immunosuppressive effects of MSCs in the lungs did not require MSCs to retain survival (135). Moreover, in myelin oligodendrocyte glycoprotein-induced experimental autoimmune encephalitis models, BKX-MSCs (depletion of the apoptotic effectors BAK and BAX) could not reduce circulating inflammatory monocytes that infiltrated the central nervous system to cause tissue injury, compared to untreated MSCs, which suggested that apoptotic MSCs were necessary for efficient *in vivo* immunosuppression and therapeutic effect (135).

The apoptotic and efferocytosis effects of MSCs could regulate macrophage phenotype polarization. For example, a study showed that efferocytosis of adipose-derived MSCs could alter the macrophages phenotype toward regulatory and anti-inflammatory phenotype (136). Another study suggested that MSC‐derived apoptotic vesicles exerted extensive regulatory effects on macrophages at the transcription level, which contributed to macrophage polarization towards the anti‐inflammation phenotype in type 2 diabetes treatment (137). Moreover, the apoptosis and efferocytosis of MSCs could induce the alterations of metabolic and inflammatory pathway in alveolar macrophages, thus affecting immunosuppression and reducing disease severity (135). Therefore, the apoptotic and efferocytosis effects of MSCs may play a beneficial role in the treatment of ALI/ARDS by modulating the polarization of macrophage phenotype, and more preclinical and clinical studies are still needed to confirm this idea in the future.

In summary, MSCs may regulate macrophage polarization through mechanisms such as paracrine soluble factors, exosomes, modulation of metabolic reprogramming and mitochondrial transfer, apoptotic and efferocytosis effects (**Table 1**). The regulation of macrophage polarization by MSCs may be a potential and promising therapy for the treatment of ALI/ARDS.

**Table 1:** The ways MSCs regulate macrophage polarization.

Authors	Publication time	Sources of MSCs	Regulation of macrophage polarization	Results	Reference
Wakayama H et al.	2015	Dental pulp	Paracrine soluble factors	Dental pulp stem cells-secreted factors could attenuate bleomycin-induced pro-inflammatory response and induce anti-inflammatory M2-like lung macrophage.	(82)
Chen et al.	2018	Dental follicle	Paracrine soluble factors: TGF-β3 and TSP-1	Dental follicle stem cells-secreted TGF-β3 and TSP-1 could not only attenuate histopathological damage and pulmonary permeability, but also downregulate pro-inflammatory cytokines, upregulate anti-inflammatory cytokine, and reprogram macrophages into the anti-inflammatory phenotype.	(72)
Kwon J H et al.	2019	Umbilical cord blood	Paracrine soluble factors: decorin	Decorin secreted by MSCs is a key modulator of macrophage polarization to regulate anti-inflammatory reactions, thus playing a protective effect on hyperoxia induced lung injury.	(83)
Kim M et al.	2020	Umbilical cord blood	Paracrine soluble factors: PTX3	MSCs-secreted PTX3 could reinforce the anti-inflammatory macrophage marker, and exert therapeutic effects in a neonatal hyperoxic lung injury	(84)
Ionescu L et al.	2012	Bone marrow	Paracrine soluble factors: insulin-like growth factor.	MSCs-secreted soluble factors could promote the resolution of LPS-induced lung injury by attenuating lung inflammation and promoting an anti-inflammatory macrophage phenotype	(85)
Németh K et al.	2009	Bone marrow	Paracrine soluble factors: Prostaglandin E2	MSCs could reprogram macrophages by releasing prostaglandin E2 and may be effective in treating sepsis	(86)
Tian et al.	2021	Adipose	MSCs-derived exosomal miR-16-5p	Exosomal miR-16-5p from adipose MSCs could promote macrophage polarization and attenuate septic lung injury in mice *via* suppressing TLR4	(114)
Song et al.	2017	Umbilical cord	MSCs-derived exosomal miR-146a	IL-1β pretreatment effectively enhanced the immunomodulatory properties of MSCs and promoted alternative macrophage polarization through exosome-mediated transfer of miR-146a	(115)
Bao et al.	2020	Bone marrow	MSCs-derived exosomal miR-21	MSCs could block pro-inflammatory pathway of macrophage through miR-21 overexpression, thus could be a potential therapeutic strategy for radiation-induced lung injury.	(116)
Li et al.	2019	Bone marrow	MSCs-derived exosomal miR-21-5p	MSCs-exosomal miR-21-5p potently reduced oxidative stress-induced apoptosis while partially reducing the pro-inflammatory, “M1” polarization of alveolar macrophage induced by hypoxia/reoxygenation.	(117)
Deng et al.	2020	Bone marrow	Metabolic reprogramming	Exosomes secreted by MSCs modulated LPS-treated macrophage polarization by inhibiting cellular glycolysis and provided novel strategies for the prevention and treatment of LPS-induced ARDS.	(63)
Morrison TJ et al.	2017	Bone marrow	Mitochondrial transfer	MSCs promoted an anti-inflammatory and highly phagocytic macrophage phenotype through EV-mediated mitochondrial transfer, thus protecting against endotoxin induced lung injury.	(73)
Xia et al.	2022	Adipose	Mitochondrial transfer	MSCs-exosomes can effectively donate mitochondria component improved macrophages mitochondrial integrity and oxidative phosphorylation level, leading to the resumption of metabolic and immune homeostasis of macrophages and mitigating lung inflammatory pathology	(127)
Ghahremani Piraghaj M et al.	2018	Adipose	Efferocytosis of MSCs	Efferocytosis of AD-MSCs can alter the macrophages phenotype toward regulatory and anti-inflammatory phenotype.	(136)
Zheng et al.	2021	Bone marrow	MSCs-derived apoptotic vesicles	MSCs-derived apoptotic vesicles could induce macrophage reprogramming at the transcription level in an efferocytosis-dependent manner, leading to inhibition of macrophage accumulation and transformation of macrophages towards an anti-inflammation phenotype, thus alleviating type 2 diabetes phenotypes including glucose intolerance and insulin resistance.	(137)

## 5 Signaling Pathways Associated With the Regulation of Macrophage Polarization by MSCs

### Nuclear Factor-Kappa B (NF-kB) Signaling Pathway

The NF-kB pathway is widely recognized as a pro-inflammatory signaling pathway that participates in host immune response. The activation of inflammatory stimuli such as LPSs leads to the release and nuclear translocation of NF-kB (138, 139). The NF-kB signaling pathway plays an important role in the regulation of macrophage polarization (140, 141).

MSCs can regulate macrophage polarization through NF-kB signaling. Gao et al. demonstrated that co-culturing LPS-induced mice macrophages with MSCs decreased TNF-α expression and enhanced IL-10 and Arg-1 expression; the authors also found that the activity of the NF-kB pathway was inhibited in MSC-treated mice macrophages (142). Wang et al. reported that MSC-derived extracellular vesicles regulated macrophage polarization, attenuated lung injury, and improved pulmonary function by inhibiting the activation of the NF-kB pathway (143). MSCs also attenuated burn-induced ALI by inhibiting the activation of the TLR4/NF-kB signaling pathway *via* paracrine TSG-6 and transforming macrophages from the pro-inflammatory phenotype to the anti-inflammatory phenotype (144). In a multidrug-resistant *Pseudomonas aeruginosa*-induced ALI mice model, MSC-derived extracellular vesicles modulated the balance between pro-inflammatory and anti-inflammatory macrophages by inhibiting the activity of the NF-kB signaling pathway, leading to the downregulation of the pro-inflammatory macrophage markers iNOS and IL-12 and the upregulation of the anti-inflammatory macrophage markers Arg-1 and IL-10; these effects reduced the bacterial load and inflammatory response, resulting in decreased mice mortality (145).

### Notch Signaling Pathway

Notch signaling is a conserved and important signal transduction pathway that mediates intercellular communication and plays a role in many cell types and different stages of development (146). The Notch signaling pathway is closely associated with inflammation and immune function (147). The Notch signaling pathway also plays a key role in the activation of macrophage polarization. The activation of the Notch signaling pathway promotes pro-inflammatory macrophage polarization and increases the expressions of pro-inflammatory macrophage markers (148–150). Bai et al. found that adipose MSC-derived extracellular vesicles regulated LPS-induced pro-inflammatory polarization by inhibiting the Notch signaling pathway and attenuating sepsis-induced lung injury and inflammatory cell infiltration in lung tissue (151).

### Nuclear Factor Erythroid 2-Related Factor 2 (Nrf2)/heme Oxygenase-1 (Ho-1) Signaling Pathway

Nrf2 is a transcription factor that regulates oxidation and inflammation to prevent oxidative damage and modulate apoptosis. Several anti-inflammatory factors can promote the nuclear translocation of Nrf2 and increase the expression of downstream HO-1 protein (152). The activation of the Nrf2/Ho-1 signaling pathway inhibits oxidative stress and inflammatory responses in sepsis-induced organ injury (153). The Nrf2/HO-1 signaling pathway plays a crucial role in regulating macrophage polarization (141, 154, 155). MSCs exert anti-inflammatory effects by activating the Nrf2/HO-1 signaling pathway, promoting anti-inflammatory macrophage polarization, inhibiting oxidative stress, and facilitating lung injury repair, which may be related to the expression of stanniocalcin-2, which has antioxidant properties, in MSCs (71).

### JAK/STAT Signaling Pathway

The Janus family of kinases (JAK) is composed of four main members, JAK1, JAK2, JAK3 and Tyk2, which is non-receptor protein tyrosine kinase by nature (156, 157). JAK family kinases can phosphorylate signal transducers and activators of transcription (STATs) to regulate the expression of related genes; this pathway is called the JAK-STAT signaling pathway, The JAK-STAT signaling pathway can modulate cell proliferation, differentiation, apoptosis, and immune function (158). The STAT family includes seven members (STAT1, STAT2, STAT3, STAT4, STAT5A, STAT5B, and STAT6) that play crucial roles in many cellular functions (159, 160). STAT1 is the most important mediator of the IFN-γ-induced polarization of pro-inflammatory macrophages (44, 161, 162). The inhibition of JAK/STAT1 signaling can attenuate the activation of pro-inflammatory macrophages (163–165). STAT3 is an important transcription factor that activates macrophages and increases inflammation (166–168). STAT6 is a major transcription factor for IL-4- or IL-13-mediated signaling and plays an important role in anti-inflammatory macrophage polarization (169). MSCs can modulate macrophage polarization by regulating the JAK/STAT pathway to reduce ulcerative colitis and obesity-associated metabolic disorders (170, 171). MSCs also play an important role in improving ALI/ARDS by regulating macrophage polarization through STAT signaling. Xu et al. found that UC-MSCs promoted anti-inflammatory polarization and T-regulatory cell differentiation to suppress inflammatory responses and reduce LPS-stimulated lung injury (50). Meanwhile, the authors found that transfection with surfactant protein B enhanced the repair of ALI/ARDS by MSCs, which may be related to the regulation of STAT3 signaling (50).

## 6 Conclusions

In summary, MSCs can regulate macrophage polarization *via* the paracrine secretion of soluble factors, exosomes, metabolic reprogramming, and mitochondrial transfer to reduce lung inflammation and promote the repair of damaged lung tissue in ALI/ARDS. The regulation of macrophage polarization may become a therapeutic target for ALI/ARDS, thereby providing a new research direction for ALI/ARDS treatment. However, there are still some unresolved issues. For example, the signaling pathways involved in the modulation of macrophage polarization by MSCs are not fully understood, and it is not clear how to prevent lung fibrosis caused by excessive M2 macrophage polarization. Therefore, the treatment of lung injury *via* the regulation of macrophage polarization requires further study to lay the foundation for clinical application.

In addition, at present, severe acute respiratory syndrome coronavirus 2 (SARS-CoV-2) infection has led to a global pandemic, MSCs therapy seems to be an alternative option to improve inflammation, repair lung tissue damage and prevent long-term pulmonary dysfunction in patients with COVID-19, and cell therapy with reprogrammed macrophages for COVID-19-induced-ARDS may be successful. For example, shifting macrophage polarization from a pro-inflammatory phenotype to an anti-inflammatory phenotype could attenuate the production of pro-inflammatory cytokines, thereby preventing cytokine storm and reducing mortality in patients with COVID-19.

## Author Contributions

The first draft of the manuscript was written by CL, and the manuscript was revised by KX and LX. All authors contributed to the article and approved the submitted version.

## Funding

This work was supported by China PLA Scientific Key Grant (18CXZ026).

## Conflict of Interest

The authors declare that the research was conducted in the absence of any commercial or financial relationships that could be construed as a potential conflict of interest.

## Publisher’s Note

All claims expressed in this article are solely those of the authors and do not necessarily represent those of their affiliated organizations, or those of the publisher, the editors and the reviewers. Any product that may be evaluated in this article, or claim that may be made by its manufacturer, is not guaranteed or endorsed by the publisher.
